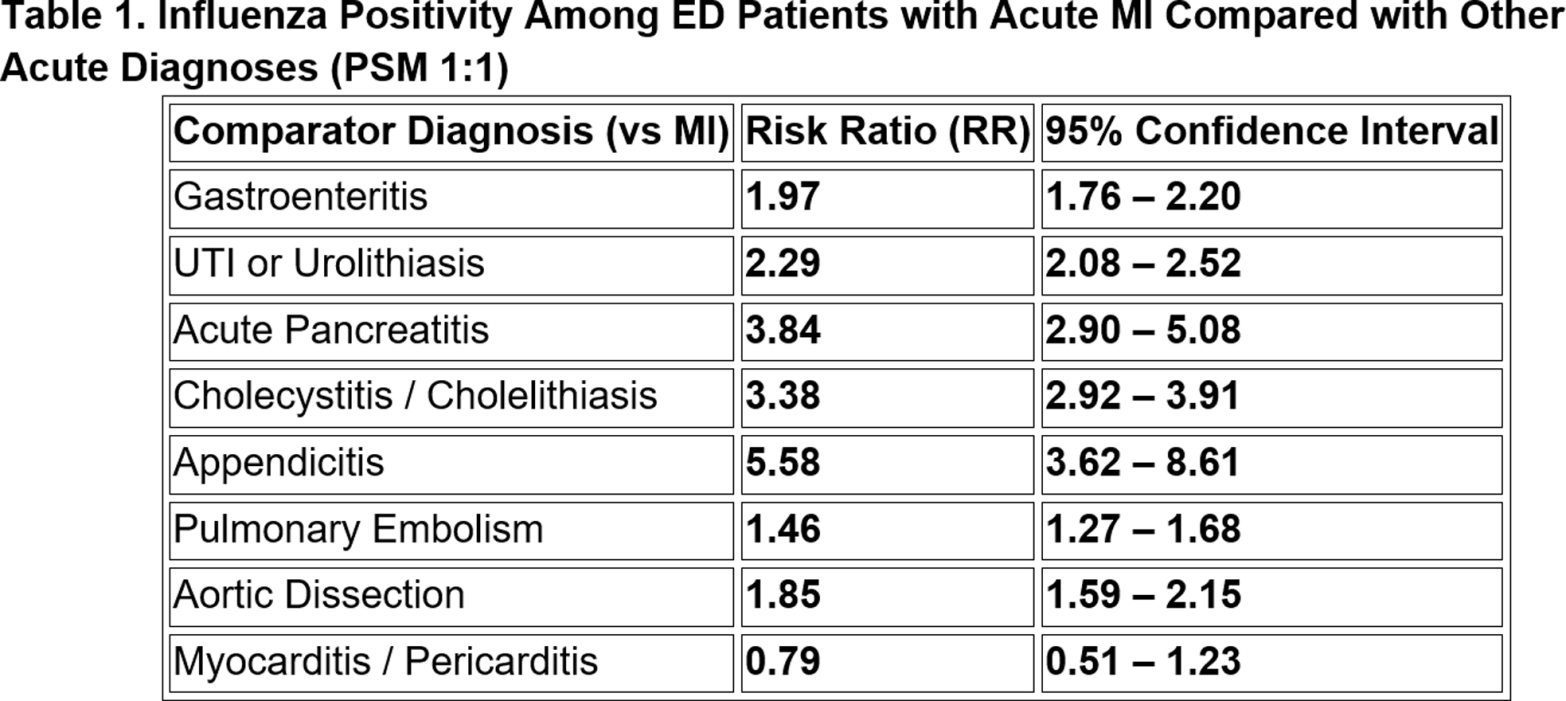# 373 Inter-Rater Agreement of Real-World Electronic Health Record-Based Antimicrobial Stewardship Assessments

**DOI:** 10.1017/ash.2026.10709

**Published:** 2026-06-23

**Authors:** Alexia El Khoury, Joy Abou Farah, Luke Kabbara, Zainab Albar, Jay Krishnan, Elie Saade, University Hospitals Cleveland Medical Center

**Affiliations:** 1 Case Western Reserve University; 2 Case Western Reserve University/ UH Hospitals; 3 University Hospitals, Case Western Reserve University

## Abstract

**Background:** Influenza infection has been temporally associated with acute cardiovascular events, including myocardial infarction (MI). We evaluated whether laboratory-confirmed influenza infection is disproportionately present among ED patients presenting with acute MI compared with patients presenting with other common acute diagnoses. **Methods:** We conducted a retrospective cohort study using TriNetX data from the 2024–2025 influenza season. Adult ED patients (<18yrs) presenting with acute MI were compared to patients presenting with mutually exclusive acute diagnoses, including gastroenteritis, urinary tract infection or urolithiasis, appendicitis, pancreatitis, cholecystitis, pulmonary embolism, aortic dissection, and myocarditis or pericarditis. Patients with COVID infection 30 days prior the ED visit were excluded. Influenza positivity during the ED encounter was defined as the outcome. All comparisons were adjusted using 1:1 propensity score matching for demographics, cardiovascular risk factors, chronic respiratory disease, and healthcare utilization indicators. Risk ratios (RRs) and 95% confidence intervals (CIs) were calculated. **Results:** Across propensity score–matched comparisons, influenza infection was consistently more prevalent among ED patients presenting with acute MI than among most comparator diagnoses. Influenza positivity was significantly higher in MI compared with gastroenteritis (RR 1.97, 95% CI 1.76–2.20), urinary tract infection or urolithiasis (RR 2.29, 95% CI 2.08–2.52), pancreatitis (RR 3.84, 95% CI 2.90–5.08), cholecystitis or cholelithiasis (RR 3.38, 95% CI 2.92–3.91), and appendicitis (RR 5.58, 95% CI 3.62–8.61). Among high-acuity cardiovascular chest-pain presentations, influenza positivity remained higher in MI compared with pulmonary embolism (RR 1.46, 95% CI 1.27–1.68) and aortic dissection (RR 1.85, 95% CI 1.59–2.15). In contrast, no significant difference was observed between MI and myocarditis or pericarditis (RR 0.79, 95% CI 0.51–1.23). **Conclusions:** In this diagnosis-based, propensity score–matched ED analysis, laboratory-confirmed influenza infection was disproportionately prevalent among patients presenting with acute myocardial infarction compared with a broad range of other acute ED diagnoses. The persistence of this association across infectious, surgical, and high-acuity cardiovascular comparators, alongside the absence of association with myocarditis or pericarditis, suggests that the observed association is not simply due to patients being sicker or undergoing more intensive diagnostic evaluation. These findings are hypothesis-generating and support further investigation into temporal and mechanistic links between influenza infection and acute coronary events.

**Figure:**